# Antimicrobial efficacy and compatibility of solid copper alloys with chemical disinfectants

**DOI:** 10.1371/journal.pone.0200748

**Published:** 2018-08-10

**Authors:** Katrin Steinhauer, Sonja Meyer, Jens Pfannebecker, Karin Teckemeyer, Klaus Ockenfeld, Klaus Weber, Barbara Becker

**Affiliations:** 1 Research & Regulatory Affairs, Schülke & Mayr GmbH, Norderstedt, Germany; 2 Ostwestfalen-Lippe University of Applied Sciences, Department Life Science Technologies, Microbiology, Lemgo, Germany; 3 Environment and Health, Deutsches Kupferinstitut Berufsverband e.V., Düsseldorf, Germany; Institute of Materials Science, GERMANY

## Abstract

**Introduction:**

Chemical disinfection is state of the art in preventing spread of infectious agents in the healthcare setting. Additionally, the antimicrobial properties of solid copper alloy surfaces against various microorganisms have recently been substantiated. Thus, antimicrobially active copper surfaces may serve as an additional barrier against distribution of pathogenic microorganisms and be combined with chemical disinfection measures in the hospital. The aim of this study was therefore to investigate on a quantitative basis whether the combination of chemical disinfectants with copper alloy surfaces results in an overall compromised, combined or even synergistic antimicrobial efficacy.

**Methods:**

Experiments were carried out using the quantitative carrier test devised by the German Society for Hygiene and Microbiology (DGHM) to study antimicrobial efficacy of chemical disinfectants. Requirements for microbicidal efficacy as defined by prEN 14885 were applied. The chemical disinfectants tested in our study contained alcohols (ethanol, 1-propanol), quaternary ammonium compounds (benzalkonium chloride) and glutaraldehyde as actives. Quantitative carrier tests were carried out on different carriers (tiles, copper alloy discs, stainless steel discs) using *Pseudomonas aeruginosa*, *Staphylococcus aureus*, *Kocuria rhizophila* and *Candida albicans* as test organisms.

**Results:**

For the alcohol-based disinfectant no difference in antimicrobial efficacy was observed when applied to antimicrobial active copper alloy carriers, tiles or stainless steel discs. For all test organisms microbial contamination was reduced to the detection limit of < 1 log (CFU/ml) within a contact time of 2 min indicating a ≥ 5 log reduction for the tested bacteria and a ≥ 4 log reduction for the yeast, as being requested for chemical disinfectants by prEN 14885. In order to elucidate a potential synergism the chemical disinfectant based on quaternary ammonium compounds (benzalkonium chloride) and glutaraldehyde was used at a sub-effective concentration. Hence, no complete reduction of microbial contamination was achieved on stainless steel or tile carriers for *Pseudomonas aeruginosa* and *Candida albicans*. Interestingly, when using copper alloy carriers complete reduction indicating a ≥ 5 log reduction for *P*. *aeruginosa* and a ≥ 4 log reduction for *C*. *albicans* was detected. Thus, data of this study indicates that solid copper alloy surfaces and disinfectants synergize.

**Conclusions:**

According to this data, commercially available disinfectants based on alcohol, quaternary ammonium compounds and aldehyde can effectively be combined in a dual strategy with solid copper alloy surfaces to reduce microbial contamination.

## Introduction

Nosocomial infections pose a great threat in the hospital setting, which is reflected in the practical guide to prevention of hospital-acquired infections published in 2012 by WHO [1]. Based on this WHO guide, factors influencing the development of nosocomial infections are microbial agents, patient susceptibility, environmental factors, and bacterial resistance [1]. Along with other aspects, objects contaminated by microorganisms are regarded as a potential source in the development of nosocomial infections [1]. Thus, effective hygiene measures are needed to prevent spread of microbial contaminants and chemical disinfectant measures are regarded as state of the art. Regarding antimicrobial efficacy of chemical disinfectants the draft version of European standard (prEN) 14885 summarizes the application of European standards as a methodological framework for chemical disinfectants and antiseptics [2]. In these tests, biocidal formulations are being evaluated using suspension and/or carrier tests that simulate practical conditions, and the antimicrobial efficacy of disinfectants is determined quantitatively by logarithmic reduction factors. Requirements for microbicidal efficacy as defined by prEN 14885 are ≥ 5 log RF for bacteria and ≥ 4 log RF for *Candida albicans* for chemical disinfectants [2].

In addition to chemical disinfectants, solid copper and copper alloys such as special brasses have been widely studied with regard to their antimicrobial efficacy during the past 10 years [3,4], and antimicrobial properties of solid copper alloy surfaces against various microorganisms have recently been substantiated.

Zhu et al. [5] provided an insight into the efficacy of different copper alloy surfaces against Gram-negative bacteria such as *Salmonella enterica*. Further studies from other research groups underline the efficacy of copper alloy surfaces against Gram-positive bacteria such as *Enterococcus* spp. and *Staphylococcus* spp., as well as non-enveloped viruses such as hepatitis A virus and norovirus [6–9]. In addition, studies on application of continuously active antimicrobial copper surfaces in hospitals could demonstrate reduction of microbial burden on touch surfaces [10, 11].

Thus when looking at effective hygiene strategies the question arises, as to whether the combination of both measures (antimicrobial copper surfaces and chemical disinfectants) may be used complementarily or even synergistically. Airey and Verran [12] examined the cleaning properties of copper surfaces after bacterial soiling with *Staphylococcus aureus* and concluded that denatured ethanol (70%) or 1% sodium hypochlorite, when applied to copper surfaces, appeared to react with copper. Based on their observations, the authors recommended further investigations on appropriate cleaning and disinfection practices. Kawakami et al. [13,14] investigated the effects of sodium hypochlorite and ethanol on cleaning properties and the re-establishment of antibacterial activities of copper-alloyed stainless steel.

However, in none of these studies was the antimicrobial efficacy of the applied chemical disinfectants evaluated quantitatively, nor was the antimicrobial efficacy of the solid copper alloys. Furthermore, Molteni et al. [15] demonstrated that killing of microbial cells on solid copper alloys is impacted by liquid medium composition, and Warnes and Keevil [6,7] provided evidence that copper ion release is affected in the presence of chelating agents (e.g. EDTA), resulting in reduced killing rates. Thus, question arises as to whether the combination of chemical disinfectants with solid copper alloy surfaces will result in an overall compromised or synergistic antimicrobial efficacy. The goal of this study was therefore to elucidate, based on quantitative efficacy tests simulating practical conditions, whether the combination of antimicrobial solid copper surfaces and chemical disinfectants results in compromised, combined or even synergistic efficacy and thus might help to build a synergistic barrier against distribution of pathogenic microorganisms. In order to address this question, the quantitative carrier test devised by the German Society for Hygiene and Microbiology (DGHM) [16] was used as being the most appropriate method to study chemical disinfectants in combination with copper alloy surfaces, and acceptance criteria as defined by prEN 14885 for biocidal efficacy of chemical disinfectants were applied [2].

## Material and methods

### Strains and culture conditions

Bacterial strains used in this study were *Pseudomonas aeruginosa* ATCC 15442, *Escherichia coli* K12 NCTC 10535, *S*. *aureus* ATCC 6538, and *Kocuria rhizophila* ATCC 9341. In addition, the yeast *C*. *albicans* ATCC 10231 was used in some experiments. Microbial strains were cultivated as specified in EN 12353 [17]. Briefly, bacteria were precultivated on tryptone soya agar (TSA, 15 g/l tryptone, 5 g/l soya peptone, 5 g/l sodium chloride, 15 g/l agar) for 18–24 h at 36°C ± 1°C. For the experiments, subcultures from the respective precultures were obtained by performing one or two additional cultivation step(s) on TSA for 18–24 h at 36°C ± 1°C. The second or third subculture was used in the experiments as a working culture. The yeast *C*. *albicans* was cultivated on malt extract agar (MEA, 30 g/l malt extract, 5 g/l mycological peptone, 15 g/l agar). For precultivation purposes, *C*. *albicans* was inoculated on MEA and incubated at 30°C ± 1°C for 42–48 h. For the experiments, subcultures from the respective preculture were obtained by performing another (two) cultivation step(s) on MEA for 42–48 h at 30°C ± 1°C. The second or third subculture was used in the experiments as a working culture.

The experiments were repeated at least three times on different days and with freshly prepared cultures, unless otherwise stated.

### Microbial survival on copper alloy

Antimicrobial efficacy of copper alloy discs (alloy CuSi21Si3P, Ø 55 mm) was investigated using the quantitative carrier test involving mechanical action devised by the German Society for Hygiene and Microbiology (DGHM) method (#14) [16].

For these experiments sterilized matt-glazed surgical tiles (50 x 50 mm) and copper alloy discs (alloy CuSi21Si3P, Ø 55 mm) were used as carriers. Bacterial test suspensions were prepared by using 10 ml tryptone-NaCl solvents (0.1% tryptone and 0.85% NaCl) per petri-dish to dissolve the bacterial lawn cultivated as described above. The titer was adjusted to give 1.5–5 x 10^7^ CFU/ml.

Organic soiling was prepared as follows: 3 g bovine serum-albumin (fraction V) was dissolved in 97 ml tryptone-NaCl solvents and sterilised by membrane filtration. 3 ml sheep erythrocyte solution (e. g. Elocin-lab, Oberhausen, Germany) was added to finally give a stock-solution of 3% BSA + 3% erythrocytes. 1 ml of the organic soiling stock-solution (3% BSA + 3% erythrocytes) was added to 9 ml bacterial suspension.

Carriers were then inoculated with 0.1 ml bacterial suspension including organic soiling (0.3% BSA and 0.3% sheep erythrocytes). The inoculum was evenly distributed on the carriers using a spatula and brought to visual dryness with a max. drying time of 60 min. (i.e. t_0_).

In this method, mechanical action is simulated by spreading a 0.2 ml aliquot of either disinfectant or standardized hard-water using a glass spatula.

Preparation of standardized hard-water was as follows: 19.84 g MgCl_2_ (anhydrous) and 46.24 g CaCl_2_ (anhydrous) were dissolved and made up to 1000 ml in deionized water and sterilized to give solution A. 35.02 g NaHCO_3_ was dissolved and made up to 1000 ml in deionized water and sterilized to give solution B. Thereafter 6 ml of solution A and 8 ml of solution B were added to 600 ml sterile deionized water and made up to 1000 ml with sterile deionized water. The pH of the resulting standardized hard-water was 7.0 ± 0.2.

Thus after drying of the test suspension 0.2 ml standardized hard-water was added to the carriers as indicated (i. e. w/ hard-water) and distributed by a glass spatula At indicated intervals (i.e. t_x_) microbial survival rates were determined. For microbial recovery purposes carriers were immersed upside down into a vessel containing 10 ml tryptone NaCl solvents and sterile glass beads (ø 3–4 mm). Microbial survival rates are expressed as log CFU/ml and were determined by serial dilution steps and subsequent cultivation on TSA, as described above. Microbial survival rates are given as mean values of at least three independent experiments and are expressed as log (CFU/ml).

### Investigation of antimicrobial efficacy of chemical disinfectants on copper alloy

Antimicrobial efficacy tests were carried out using the DGHM carrier test without mechanical action and in the absence of organic soiling [16].

Carriers applied in the experiments were sterilized copper alloy discs (alloy CuZn23AlCo, 50x50 mm), stainless steel discs (alloy 1.4301, 50x50 mm) and tiles (as specified by DGHM, 50x50 mm). Preparation of microbial test suspensions (containing no organic soiling) was carried out as described above. Titers were adjusted to give 10^8^ CFU/ml, and 50 μl of microbial test suspension was used for the inoculation of test carriers. Inocula were dried for 60 or 120 min as indicated (i. e. t_0_).

Efficacy tests were carried out using an alcohol-based disinfectant (trade name mikrozid AF; 100 g contains: 25 g ethanol (94%), 35 g 1-propan-ol; Schülke & Mayr GmbH, Germany) and a formulation based on quaternary ammonium compounds and glutaraldehyde (trade name antifect extra; 100 g contains: 9.8 g glutaraldehyde, 5.0 g alkyl dimethylbenzyl ammonium chloride (C12-C16), 5.0 g didecyldimethyl ammonium chloride; Schülke & Mayr GmbH, Germany). Concentrations resulting in sub-effective microbicidal efficacy as defined by prEN 14885 (i. e. ≤ 5 log RF for bacteria and ≤ 4 log RF for *C*. *albicans*) [2] of the chemical disinfectants were chosen to be used in the experiments. After the indicated inoculum drying time (t_0_) 100 μl disinfectant was applied to the carriers and tests were carried out with a contact time of 2 min. The alcohol-based formulation (mikrozid AF) was provided as a ready-to-use product and was applied without any further dilution. The formulation based on quaternary ammonium compounds and glutaraldehyde (antifect extra), provided as a concentrate, was diluted with standardized hard-water (see above) to give a final concentration of 0.25% (v/v).

Recovery of bacteria was performed by immersion of the carriers in a vessel containing 10 ml neutralizing agent (see below) and use of a spatula. Microbial survival rates are expressed as log CFU/ml and were determined by serial dilution steps and subsequent cultivation on TSA (bacteria) or MEA (yeast), as described above. Where indicated, the level of antimicrobial efficacy was calculated according to the DGHM guideline [16]:

logarithmiccellcountdifferenceRF=log(CFUCo1)–log(CFUD),

where RF is the reduction factor and CFU Co1 and CFU D are the numbers of CFU per milliliter determined after exposure to the water control and the disinfectant, respectively. When copper alloy surfaces were used in the experiments CFU Co1 from tile carriers is used as the reference to calculate RF.

A combination of polysorbate 80 (30 g/l), lecithin (3 g/l), L-histidine (1 g/l), and sodium thiosulfate (5 g/l) in aqua bidest was demonstrated to be effective in the neutralization experiments for both biocidal formulations and was therefore used throughout the experiments.

## Results

### Evaluation of microbial survival on copper alloy

Survival of Gram-positive and Gram-negative bacteria on copper alloy was investigated using *K*. *rhizophila* and *P*. *aeruginosa* as test organisms. Even though *K*. *rhizophila* is not a typical pathogen in the hospital environment, it was chosen as an additional Gram-positive test organism to verify some data obtained with *S*. *aureus* throughout this study.

**Fig 1:**
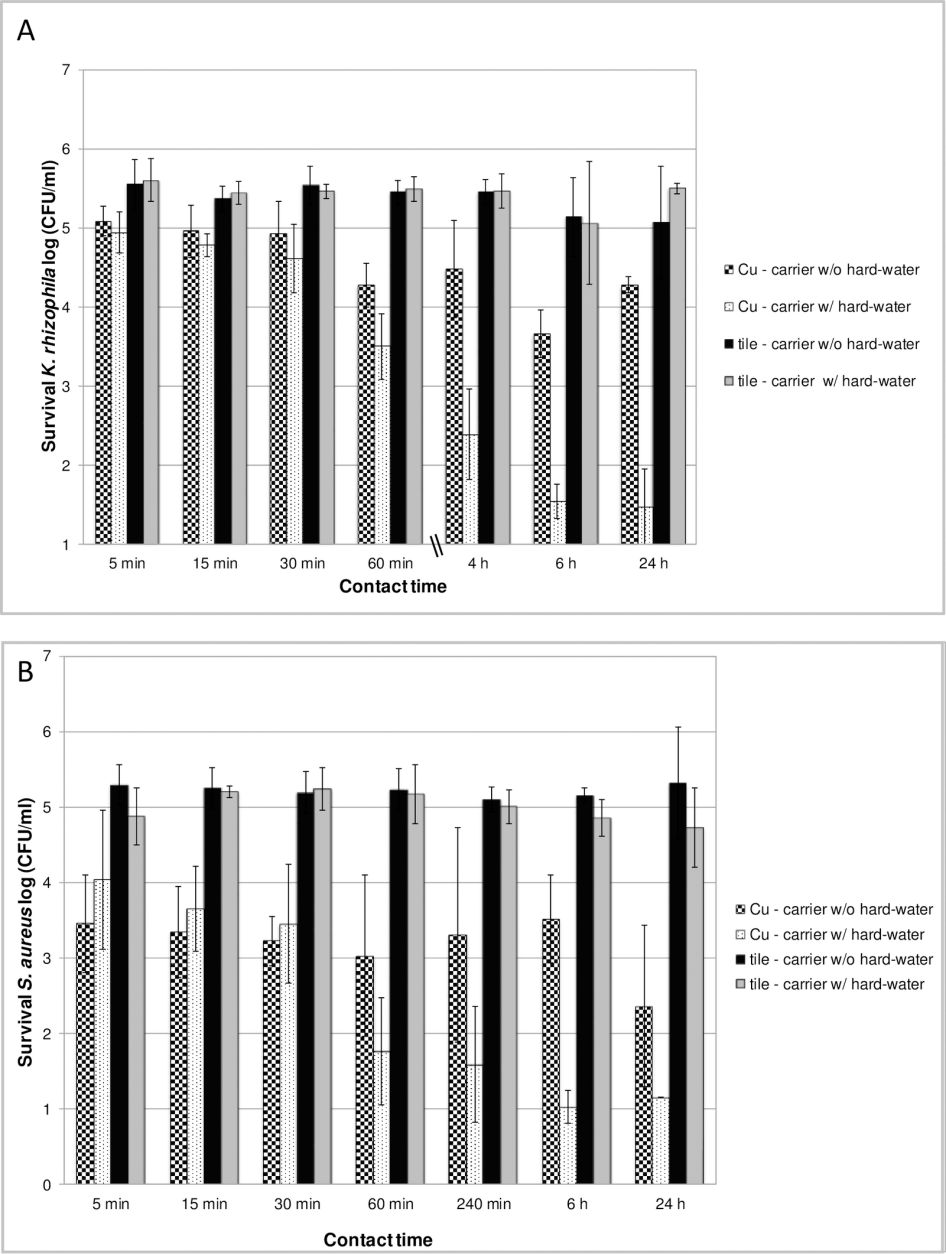
Microbial survival on copper alloy discs (CuSi21Si3P) and tile carriers. Carriers were inoculated with 100 μl bacterial suspension of *K*. *rhizophila* (titer: 1.5–5 x 10^7^ CFU/ml; **Fig 1A**) and *S*. *aureus* (titer: 2.4 x 10^7^ CFU/ml; **Fig 1B**) in the presence of organic soiling (0.3% BSA; 0.3% sheep erythrocytes). After drying for max 60 min, 200 μl standardized hard-water was added to the samples indicated as “w/ hard-water”. Samples indicated as “w/o hard-water” received no addition of standardized hard-water. Microbial survival rates were determined as described in Material and Methods at the intervals indicated and expressed as log CFU/ml. The experiments were repeated at least three times on different days. Mean values are displayed with standard error. The design of the experiments is depicted schematically in S1 Fig.

Logarithmic survival rates were determined for each test organism on copper alloy discs (alloy CuSi21Si3P, Ø 55 mm) in the presence and absence of 200 μl standardized hard-water, which was applied immediately before starting the contact time (i.e. after the inoculum has dried). Tile carriers as defined by the DGHM carrier test method [16] served as controls and were treated accordingly. Microbial survival was determined at indicated time points. Data obtained for the Gram-positive bacterium *K*. *rhizophila* is presented in Fig 1A. Survival of *K*. *rhizophila* was impacted to a greater extent on copper alloy discs than on the tile carrier after a contact time of 60 min, resulting in 4.28 ± 0.28 log and 3.50 ± 0.42 log for the copper alloy discs versus 5.45 ± 0.15 log and 5.50 ± 0.16 log for the tile carriers. Interestingly, survival of *K*. *rhizophila* and *S*. *aureus* was affected even more on copper alloy discs to which an aliquot of 200 μl hard-water had been added (Fig 1A and 1B, Cu-carriers w/ hard-water). This effect was detected at contact times ≥ 60 min.

**Fig 2:**
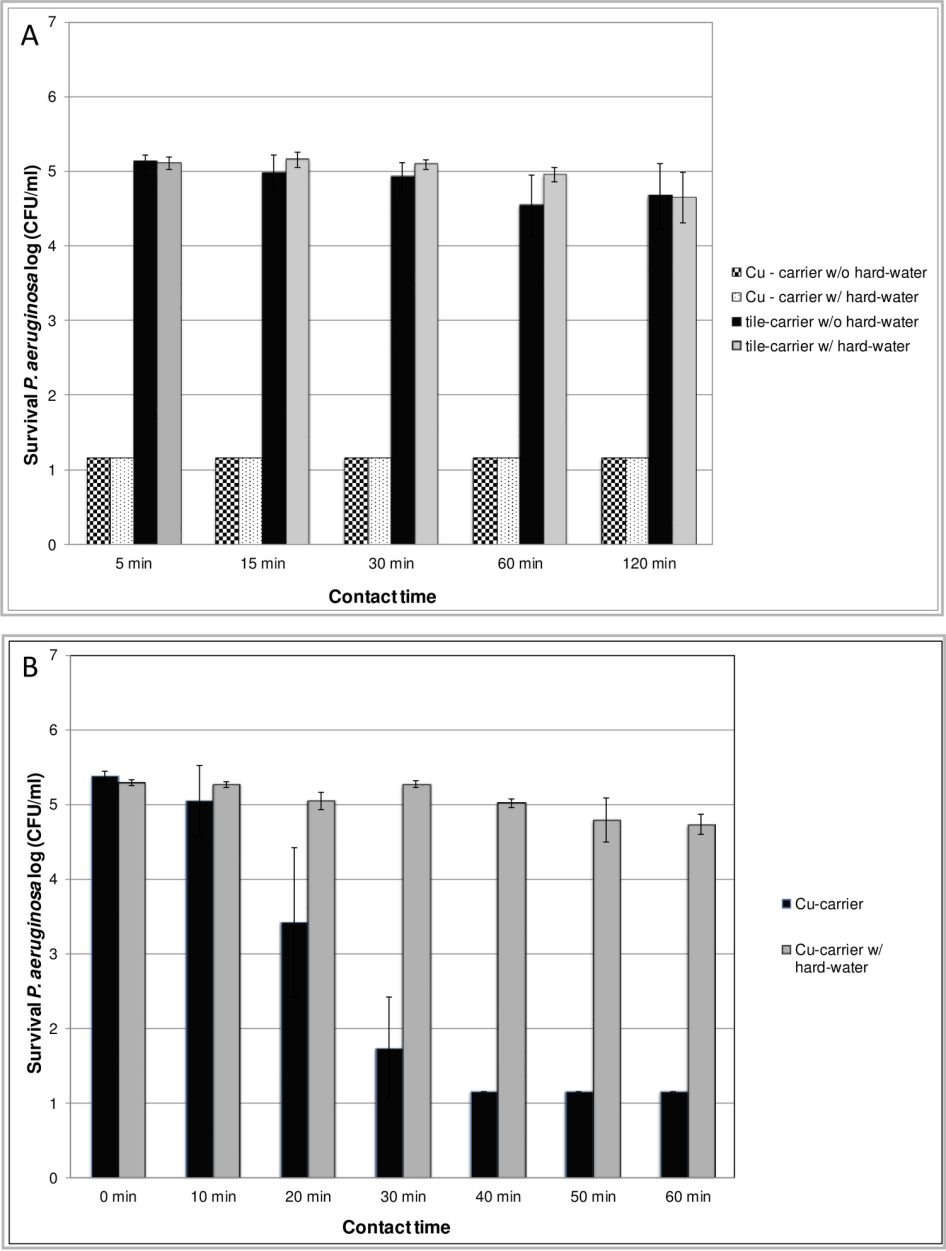
Impact of humidity on survival of *P*. *aeruginosa* on copper alloy discs (CuSi21Si3P). **A:** Carriers were inoculated with 100 μl bacterial suspension of *P*. *aeruginosa* (titer: 1.5–5 x 10^7^ CFU/ml) in the presence of organic soiling (0.3% BSA; 0.3% sheep erythrocytes). After drying for max 60 min, 200 μl standardized hard-water was added to the samples indicated as “w/ hard-water”. Samples indicated as “w/o hard-water” received no addition of standardized hard-water. Microbial survival rates were determined at the intervals indicated in Material and Methods and expressed as log CFU/ml. The design of the experiments is depicted schematically in S1 Fig. **B:** Copper alloy discs were inoculated with 100 μl bacterial suspension of *P*. *aeruginosa* (titer: 1.5–5 x 10^7^ CFU/ml) in the presence of organic soiling (0.3% BSA; 0.3% sheep erythrocytes). Immediately after inoculation, 200 μl standardized hard-water was added exclusively to the samples indicated as “w/ hard-water”. Microbial survival rates were determined as described in Material and Methods at the intervals indicated and expressed as log CFU/ml. The experiments were repeated at least three times on different days. Mean values are displayed with standard error. The design of the experiments is depicted schematically in S2 Fig.

Survival of the Gram-negative bacterium *P*. *aeruginosa* on the tested copper alloy discs was found to be below the detection limit of 1.15 log after only 5 min of contact time in the absence or in the presence of a 200 μl aliquot of standardized hard-water (Fig 2). In contrast, survival of *P*. *aeruginosa* on tile carriers ranged from > 5 log (5 min contact time) to > 4.5 log (120 min contact time) when tested with or without hard-water. The DGHM carrier test requires drying of the inoculum within a maximum of 60 min prior to determination of efficacy at defined contact times [16]. Thus, the data presented in Fig 2A suggest that the tested copper alloy discs already exerted an effect on survival of *P*. *aeruginosa* within the inoculum drying period. This observation led to the experiments presented in Fig 2B, where survival of *P*. *aeruginosa* was determined at intervals of 10 min starting immediately after the inoculum had been placed on the copper alloy discs in the presence or absence of 200 μl hard-water. Survival of *P*. *aeruginosa* was found to remain stable on copper alloy discs in the presence of 200 μl hard-water. However, survival of *P*. *aeruginosa* was reduced to the detection limit of ≤ 1.15 log after 40 min in those samples where no standardized hard-water had been added. This finding is consistent with the data presented in Fig 2A, as according to the methodology of the DGHM carrier test no hard-water is to be added within the drying period.

### Antimicrobial efficacy of an alcohol-based disinfectant on copper alloy

**Fig 3:**
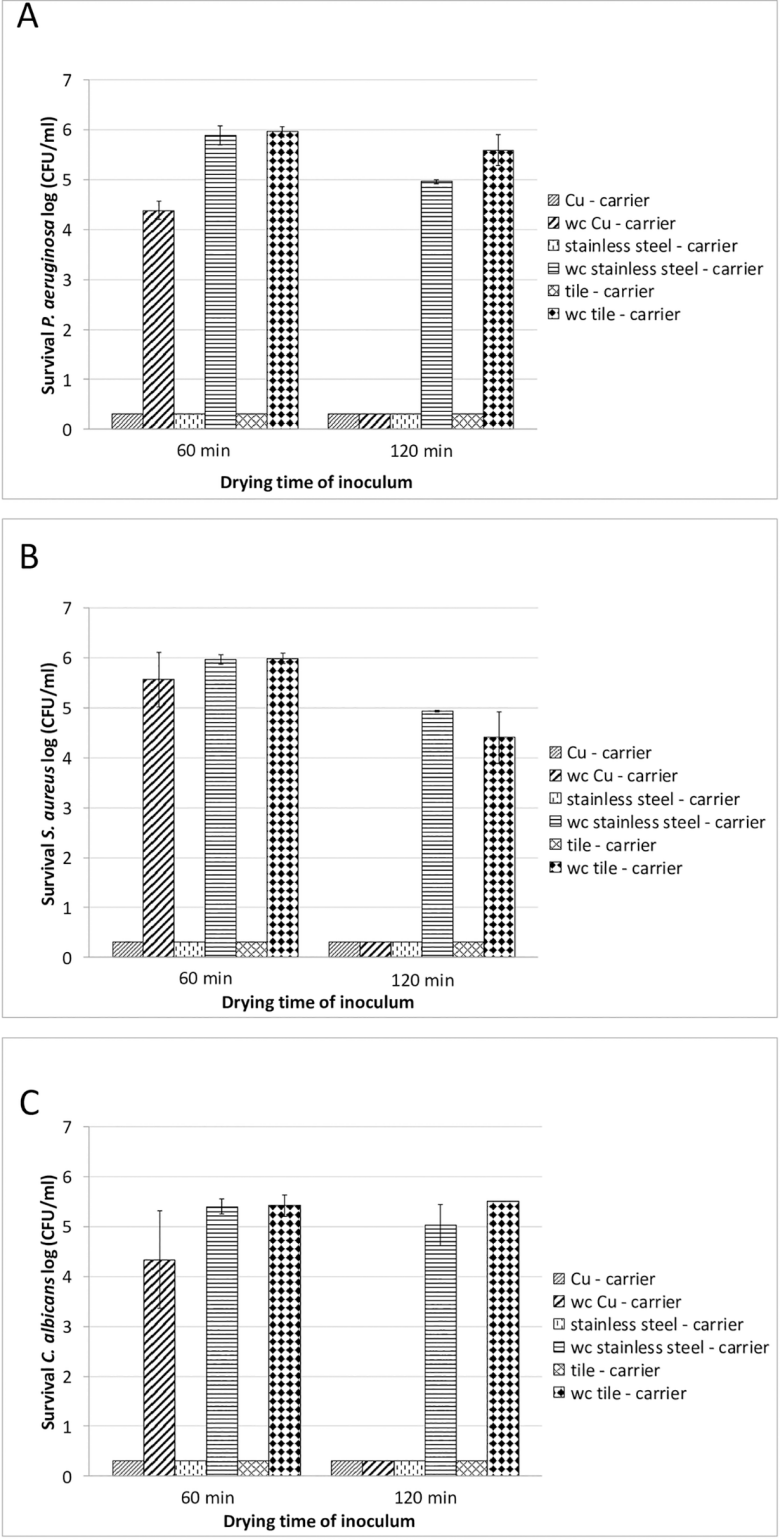
Investigation of antimicrobial efficacy of an alcohol based-disinfectant on copper alloy (CuZn23AlCo). Antimicrobial efficacy tests were carried out using the DGHM carrier test without mechanical action and without organic soiling [16]. Microbial inocula were dried for either 60 min or 120 min, as indicated. 100 μl disinfectant was applied to the different carriers (copper alloy carriers, stainless steel carriers, tile carriers) with a contact time of 2 min. Water controls (wc) were used to observe the influence of the different carriers (application of 100 μl standardized hard-water instead of the disinfectant within the contact time). Microbial survival rates were determined as described in Material and Methods at the intervals indicated and expressed as log CFU/ml. **A: Test organism *P*. *aeruginosa*. B: Test organism *S*. *aureus***. **C: Test organism *C*. *albicans***. The experiments were repeated at least three times on different days. Mean values are displayed with standard error. The design of the experiments is depicted schematically in S3 Fig.

The antimicrobial efficacy of an alcoholic formulation containing 35 g 1-propan-ol and 25 g ethanol per 100 g (mikrozid AF, Schülke & Mayr GmbH) was tested on different carrier materials (tiles, stainless steel discs, and copper alloy discs) as described in Material and Methods. The test organisms used were *P*. *aeruginosa*, *S*. *aureus*, and the yeast *C*. *albicans*. Inocula were dried on the carriers for either 60 or 120 min prior to application of the alcoholic disinfectant. Experiments were conducted without organic soiling at a contact time of 2 min. Fig 3A–3C show that no difference in the antimicrobial efficacy of the alcoholic formulation could be observed when comparing the three different carriers. For all test organisms and all test surfaces, microbial contamination was reduced to the detection limit < 1 log in the presence of the alcoholic biocide. Thus no impairment of the biocidal efficacy of the disinfectant could be observed when using the alcohol-based formulation on either test carrier. Interestingly, when looking at the water controls (application of 100 μl standardized hard-water instead of the disinfectant within the contact time), microbial soiling was also reduced to the detection limit of < 1 log when drying of the inoculum was extended to 120 min where copper alloy discs had been used as the carrier material.

### Antimicrobial efficacy of a disinfectant based on quaternary ammonium compounds and aldehyde on copper alloy

**Fig 4:**
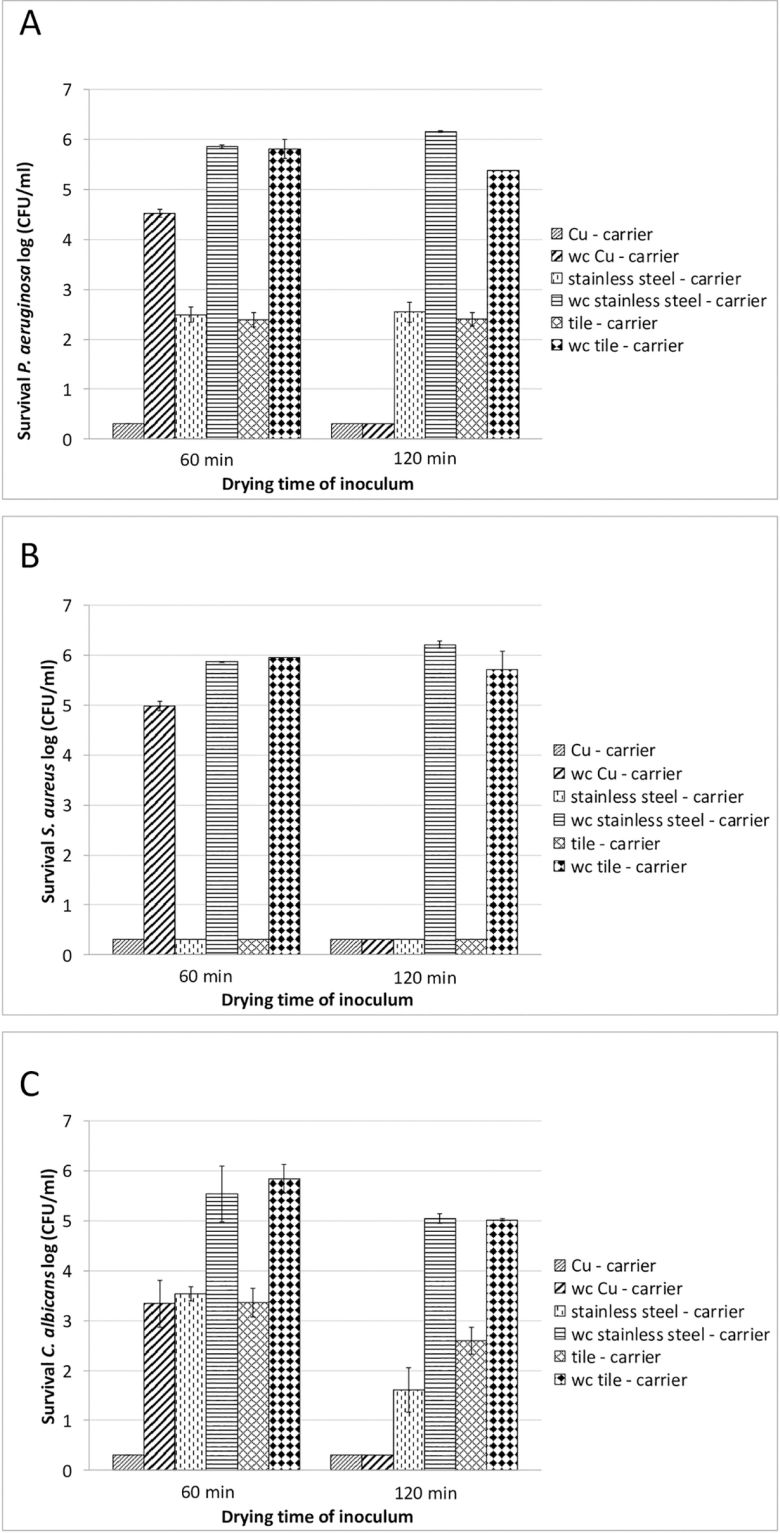
Investigation of antimicrobial efficacy of a quaternary ammonium compound-based disinfectant on copper alloy (CuZn23AlCo). Antimicrobial efficacy tests were carried out using the DGHM carrier test with and without organic soiling [16]. Microbial inocula were dried for either 60 min or 120 min, as indicated. 100 μl disinfectant dilution (diluted to give 0.25% (v/v)) was applied to the different carriers (copper alloy carriers, stainless steel carriers, tile carriers) with a contact time of 2 min. Water controls (wc) were used to observe the influence of the different carriers (application of 100 μl standardized hard-water instead of the disinfectant within the contact time). Microbial survival rates were determined as described in Material and Methods at the intervals indicated and expressed as log CFU/ml. **A: Test organism *P*. *aeruginosa*. B: Test organism *S*. *aureus***. **C: Test organism *C*. *albicans***. The experiments were repeated at least three times on different days. Mean values are displayed with standard error. The design of the experiments is depicted schematically in S3 Fig.

Similar experiments to those described above were carried out using a biocidal formulation based on quaternary ammonium compounds and aldehyde (trade name antifect extra; 100 g contains: 9.8 g glutaraldehyde, 5.0 g alkyl (C12-16) dimethylbenzyl ammonium chloride (C12-C16), 5.0 g didecyldimethyl ammonium chloride; Schülke & Mayr GmbH, Germany). Experiments were conducted applying a sub-effective concentration of the disinfectant, which is a concentration known not to meet the efficacy requirements for chemical disinfectants based on prEN 14885(i.e. a ≥ 4 log reduction for yeast and a≥ 5 log reduction for bacteria, [2]). Thus, a concentration of 0.25% for a contact time of 2 min was used in these experiments. The data indicate that for *P*. *aeruginosa* (Fig 4A) and *C*. *albicans* (Fig 4C) no complete reduction in the presence of the biocidal formulation was achieved when using either stainless steel or tile carriers. When using copper alloy carriers, however, full reduction of *P*. *aeruginosa* (RF ≥ 5 log) and *C*. *albicans* (RF ≥ 4 log) was observed when the quaternary ammonium compound/aldehyde containing biocide was applied under these conditions. Using *S*. *aureus* as the test organism, full reduction (RF ≥ 5 log) was achieved for all test carriers in the presence of the biocide, indicating no impairment of the biocidal efficacy of the disinfectant by either carrier material (Fig 4B). Furthermore, Fig 4A and 4C show the impact of the carrier material on antimicrobial efficacy compared to the biocidal efficacy of the quaternary ammonium compound-based disinfectant. According to the data obtained for *P*. *aeruginosa* and *C*. *albicans*, an additional effect of copper alloy on antimicrobial efficacy could be demonstrated in comparison to stainless steel and tile carriers when using sub-effective concentrations of the quaternary ammonium compound-based disinfectant (Fig 4A and 4C). However, no such effect could be demonstrated in these experiments for *S*. *aureus*, as the quaternary ammonium compound-based disinfectant was found to be fully active against *S*. *aureus* under the chosen test conditions.

## Discussion

Many investigations have demonstrated that copper surfaces with a copper content of at least 55% have antimicrobial properties, and antimicrobial applications of copper have recently been reviewed by Vincent et al. [4]. Interestingly, it has been found that killing of bacteria is much faster on dry surfaces compared to wet surfaces [5–7]. However, in our experimental set-up for investigation of the antimicrobial efficacy of disinfectants on solid copper alloy, bacterial soiling was applied using 100 μl tryptone-NaCl solvents (0.1% tryptone and 0.85% NaCl). This is a requirement according to DGHM method 14, a well-established guideline for testing disinfectants [16]. Using this guideline, we examined whether contact killing is enabled under these experimental conditions. In their study using *S*. *enterica*, Zhu et al. [5] demonstrated that under wet conditions no surviving bacteria could be found after 0.5–2 h contact time when high copper content alloys were used. Using low copper content alloys under moist incubation conditions, the authors found that cell counts decreased 2–4 log. Our data show that bacterial survival was impacted by the different copper alloys within 0.5–2 h in the same order of magnitude as has been shown by Zhu et al. [5]. This indicates that the DGHM carrier test, in which drying of the solvent inoculum is a requirement, can be used to study antimicrobial efficacy of chemical disinfectants on copper alloy surfaces.

When using the Gram-negative bacterium *P*. *aeruginosa* in our experiments, no surviving bacteria (< 1.15 log) could be detected on copper alloy (CuSi21Si3P) surfaces after 60 min drying time. However, if the inoculum was kept wet within the 60 min period by addition of an aliquot of 200 μl standardized hard-water, recovery rates were 4.5–5.5 log. This corresponds to the findings of Espirito Santo et al. [18], who showed in their experiments using *E*. *coli* as the test organism that contact killing was much more pronounced on dry surfaces than on moist surfaces. The authors demonstrated that copper accumulation within the bacterial cell was much faster from dry surfaces due to the absence of buffering medium. Thus, our survival experiments with *P*. *aeruginosa* on copper alloy surfaces indicate that the inoculum has become sufficiently dry to enable the proposed mechanism of contact killing [3,6,18] when using the carrier test method according to DGHM [16] with a drying time of 60 min.

In our experiments, addition of a 200 μl aliquot of standardized hard-water resulted in higher reduction rates compared with those samples without additional solvent. This effect could be observed after the respective samples (indicated as w/ hard-water (Fig 1A) or water control (Figs 3 and 4)) had become sufficiently dry again, i.e. after approximately 60 min (Fig 1A) or 120 min (Figs 3 and 4), respectively. In their experiments using mutant strains of *E*. *hirae* unable to extrude copper Molteni et al. [15] demonstrated that ionic copper released from solid copper surfaces is an important factor in antimicrobial activity. The authors demonstrated that application of liquid media, resulting in higher copper release rates from solid copper surfaces, had proportional antimicrobial efficacy. The increased reduction rates detected in our experiments in those samples with additional solvent application could thus be explained by an increased release of ionic copper due to the additional liquid. As contact killing has been found to be most effective under dry conditions [6], it might be postulated that after the samples had become sufficiently dry to enable contact killing, the increased reduction rates that we detected result from a higher accumulation of ionic copper on the surfaces of these samples. However, further experiments are needed in order to verify this hypothesis.

Previously, most studies on the antimicrobial properties of copper alloy surfaces focused on either the killing kinetics or the applicability of copper alloys in infection prevention [7,9,10,19,20]. There are, however, only a few studies that investigate the compatibility of chemical disinfectants with copper alloys [12–14]. Using the quantitative carrier test devised by DGHM, this is the first study to investigate the impact of antimicrobial active copper alloy surfaces on chemical disinfectants and vice versa.

The data presented in this study demonstrate that efficacy of commercially available chemical disinfectants based on alcohol, quaternary ammonium compounds or aldehyde is not impacted when these disinfectants are applied to antimicrobial active copper alloy surfaces. This includes antimicrobial efficacy against Gram-positive and Gram-negative bacteria as well as yeasticidal efficacy, which was investigated in this study. Moreover, our data indicated a synergism between solid copper alloy (CuZn23AlCo) and disinfectants when looking at the data presented in Fig 4. This was specifically detectable for the Gram-negative test organism *P*. *aeruginosa* and the yeast *C*. *albicans*, whereas no such effect was detectable for the more drying-resistant Gram-positive test organism *S*. *aureus*.

In their studies on the mode of action of ionic copper, Warnes and Keevil [6,7] conclude that targets in Gram-positive and Gram-negative bacteria vary. The authors demonstrated that multifaceted events are involved in cell death and that breakdown of genomic DNA was proportionally associated with cell death. The chemical disinfectants tested in our study contained alcohols (ethanol, 1-propanol), quaternary ammonium compounds (benzalkonium chloride) and glutaraldehyde as actives. All of these biocidal substances are known for their protein denaturing properties rather than for their DNA degradation characteristics [21]. The observed synergism may thus be explained by DNA degradation due to ionic copper fostering microbial death as an additional cause.

## Conclusions

In conclusion, data presented in this study based on antimicrobial efficacy tests demonstrates that quantitative data can be obtained to elucidate the compatibility of chemical disinfectants with antimicrobial active copper alloy surfaces. No impairment of the biocidal efficacy of the tested commercially available disinfectants (based on alcohol, quaternary ammonium compounds and aldehyde) by the solid copper alloy surfaces used in these experiments was detected. The experimental set-up used in this study even revealed a synergism between solid copper alloy surfaces with a disinfectant based on quaternary ammonium compounds and aldehyde, which fosters antimicrobial efficacy. This was specifically detectable for *P*. *aeruginosa* and *C*. *albicans* when sub-effective concentrations of the disinfectant (based on the requirements of prEN 14885 [2]) were applied.

According to this data, disinfectants based on alcohol, quaternary ammonium compounds and aldehyde can be combined with solid copper alloy surfaces in a dual strategy to reduce microbial contamination on surfaces.

Nevertheless, given the rather complex mode of action of ionic copper, which has been demonstrated by different research groups [5,7,15,18], fur. ther understanding of the interaction between chemical disinfectants and microorganisms on copper alloys will require continuing investigations.

## Supporting information

S1 Fig Illustration of experimental design for data presented in Figs 1 and 2A. (TIF)

S2 Fig Illustration of experimental design for data presented in Fig 2B. (TIF)

S3 Fig Illustration of experimental design for data presented in Figs 3 and 4. (TIF)
