# Supplementary figures and images for: Antimicrobial efficacy and compatibility of solid copper alloys with chemical disinfectants
Source: PLoS One. 2018 Aug 10;13(8):e0200748. doi: 10.1371/journal.pone.0200748 (PMC6086424; doi:10.1371/journal.pone.0200748)

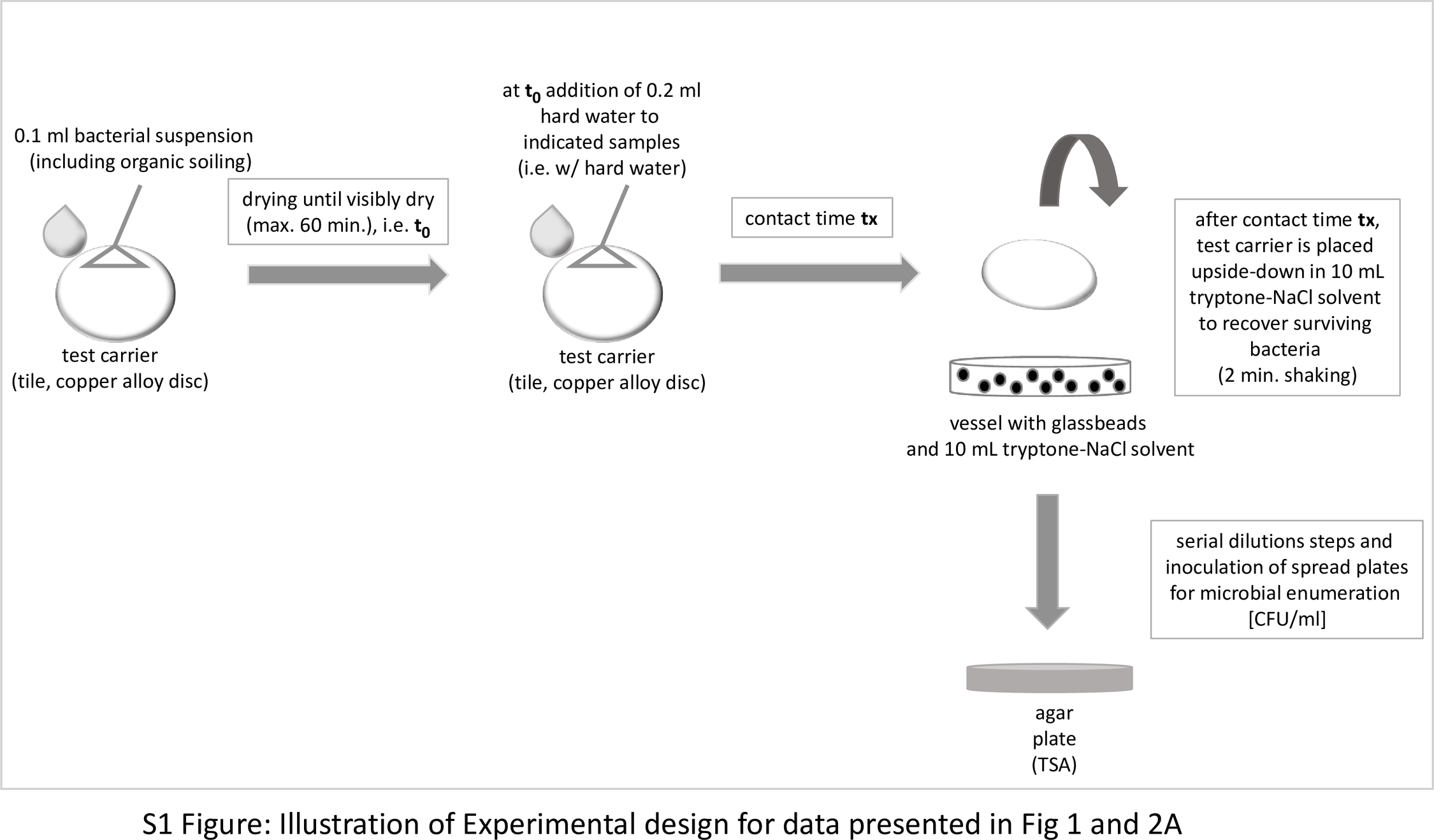

Supplement: S1 Fig — (TIF) [file pone.0200748.s001.tif]

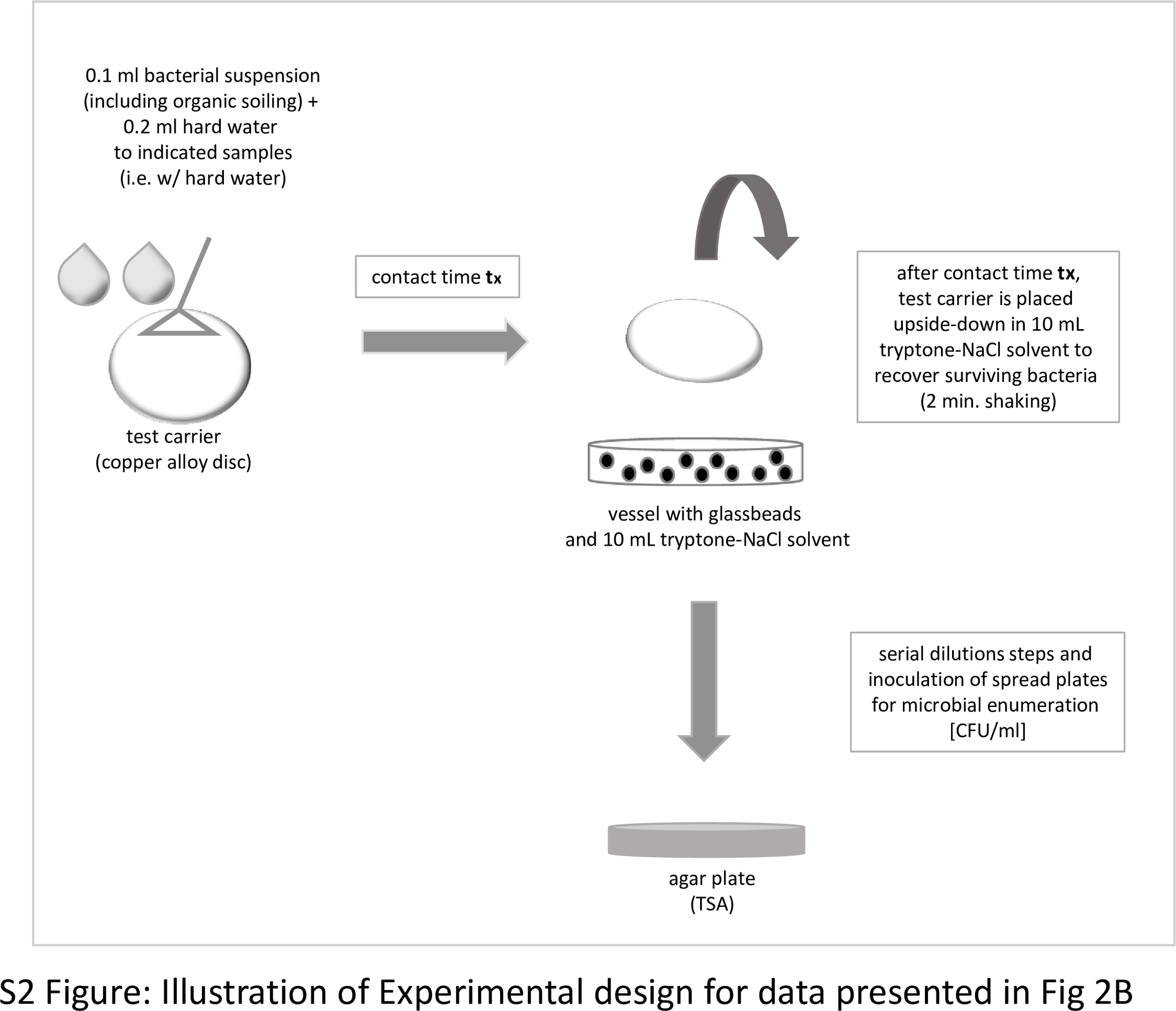

Supplement: S2 Fig — (TIF) [file pone.0200748.s002.tif]

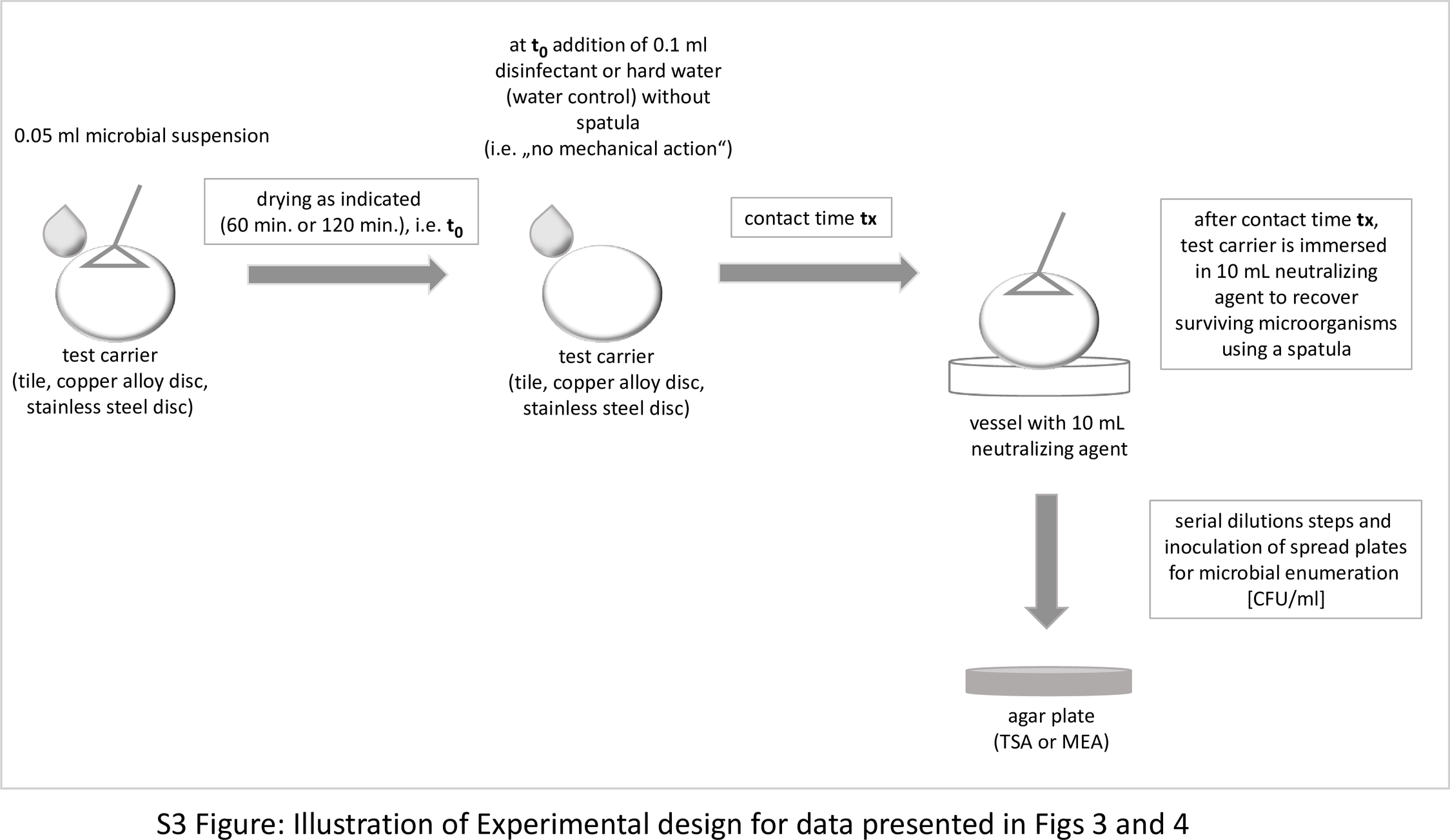

Supplement: S3 Fig — (TIF) [file pone.0200748.s003.tif]
